# Freiburg Neuropathology Case Conference: A Hearing-Impaired Patient Presenting with Lower Limb Ataxia and Progressive Personality Changes

**DOI:** 10.1007/s00062-020-00912-3

**Published:** 2020-05-25

**Authors:** C. A. Taschner, M. Schwabenland, C. Scheiwe, H. Urbach, N. Lützen, M. Prinz

**Affiliations:** 1 Department of Neuroradiology, Medical Centre, University of Freiburg, Breisacherstraße 64, 79106 Freiburg, Germany; 2 Department of Neuropathology, Medical Centre, University of Freiburg, Freiburg, Germany; 3 Department of Neurosurgery, Medical Centre, University of Freiburg, Freiburg, Germany

**Keywords:** Myxopapillary ependymoma, Paraganglioma, Hemangioblastoma, Metastasis, Superficial siderosis

## Case Report

**Fig. 1:**
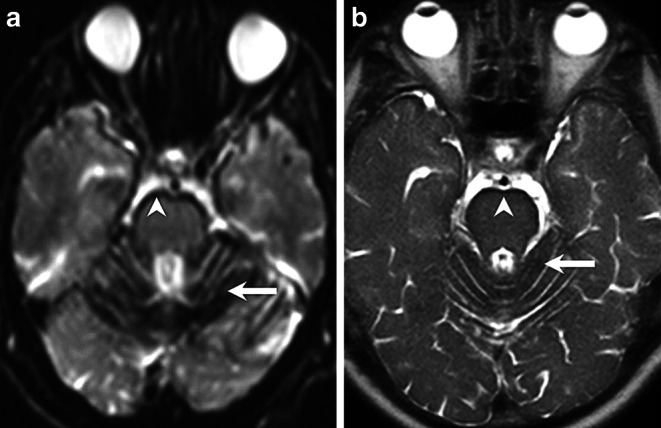
Axial diffusion-weighted (b1000, **a**) and axial T2-weighted images (**b**) show extensive superficial siderosis along the brainstem (*arrowhead*) and the cerebellar folia (*arrow*)

**Fig. 2:**
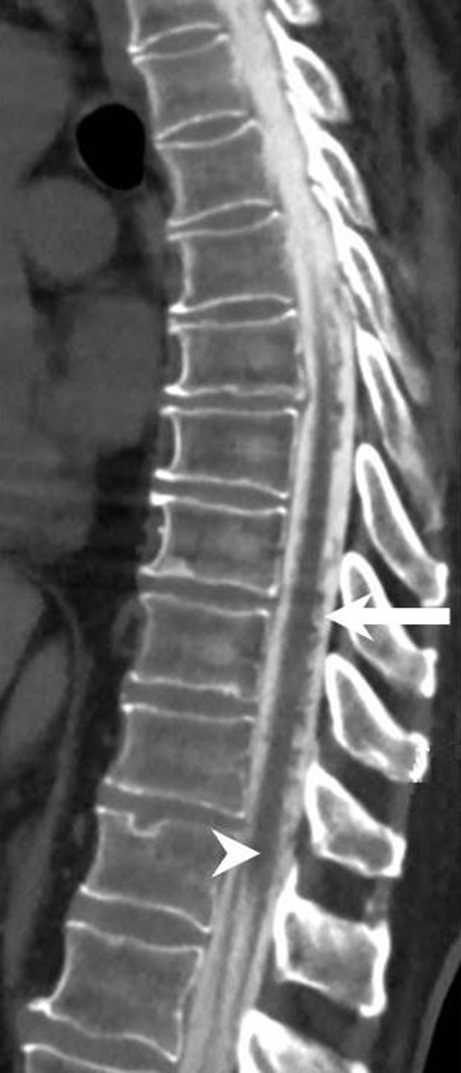
On sagittal reconstruction of a spinal CT after intrathecal administration of contrast agent a dilation of perimedullary veins (*arrow*) along the dorsal surface of the myelon through to the conus (*arrowhead*) is present

**Fig. 3:**
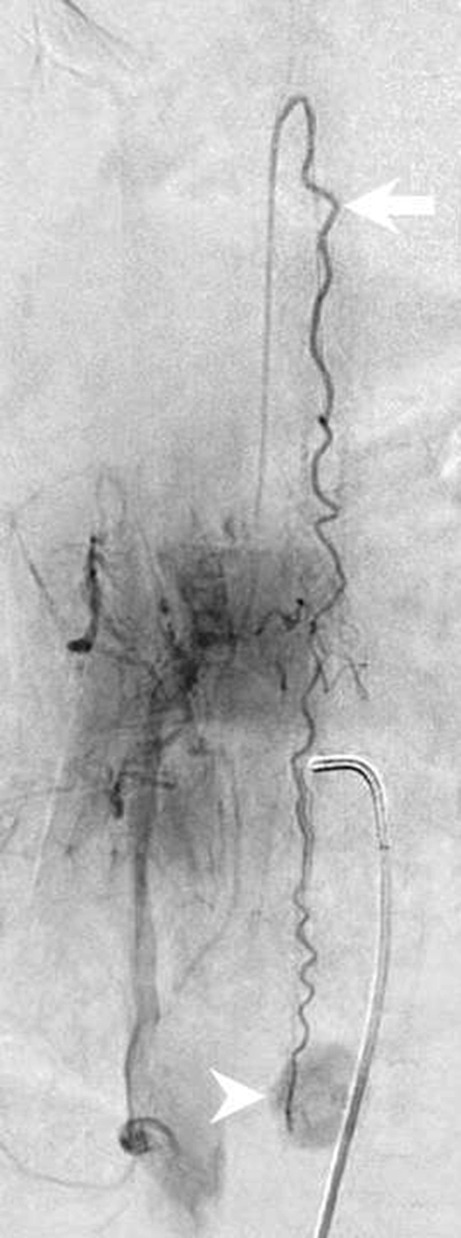
Spinal catheter angiography injected through the right lumbar artery at the L1 level shows filling of a dilated cauda equina artery (*arrow*) through a radicular anterior artery as well as a tumor blush (*arrowhead*) at the L3 level

A 57-year-old patient had presented 5 years earlier with progressive bilateral hearing loss. At the time a magnetic resonance imaging (MRI) of the head had been performed, which excluded structural abnormalities of the inner ear as well as the presence of vestibular schwannoma. In retrospect, superficial siderosis of the brainstem and the cerebellum could be detected on the corresponding MRI (Fig. 1). Since the hearing loss progressed significantly the patient was treated with a left-sided cochlear implant (CI) 2 years later. Another 2 years later the patient was hospitalized in the department of neurology for vertigo, slight tremor as well as signs of ataxia, predominantly in the left leg. In addition, the patient complained of unexplained mood swings and progressive sleepiness. Among other clinical tests a lumbar puncture was performed which revealed the presence of subarachnoid blood. An MRI of the head had to be discontinued since the patient reported pain at the level of the CI when advanced into the gantry of the MR scanner. A computed tomography (CT) and CT angiography of the head were slightly distorted due to beam hardening artifacts related to the CI but revealed no subarachnoid blood or neurovascular pathologies (not shown). Whole body positron emission tomography with different radiotracers excluded signs of cerebellar multisystem atrophy, malignancies as well as a suspected cerebral amyloid angiopathy (not shown). Since additional MRI examinations of the spine could not be performed the patient underwent a Spinal CT after intrathecal administration of an ionic contrast agent which displayed dilated perimedullary veins (Fig. 2). A spinal catheter angiography revealed a hypertrophic radiculomedullary artery and a intraspinal tumor blush at the level L3 (Fig. 3). The patient underwent a spinal MR. This time the CI was firmly fixed with a bandage and the patient was positioned feet first into the gantry of a 1.5 T MR scanner. The MRI revealed a tumor at the level of L3 of about 1.5 cm in diameter. After interdisciplinary discussion, the indications for tumor resection were confirmed. Complete resection of the highly vascularized tumor was performed via a minimally invasive dorsal approach with extended right partial hemilaminectomy of L3. After initial postoperative delirium no new focal neurological deficits were observed and the postoperative course was uneventful. Cognition and gait disturbance improved significantly during rehabilitation.

## Imaging

**Fig. 4:**
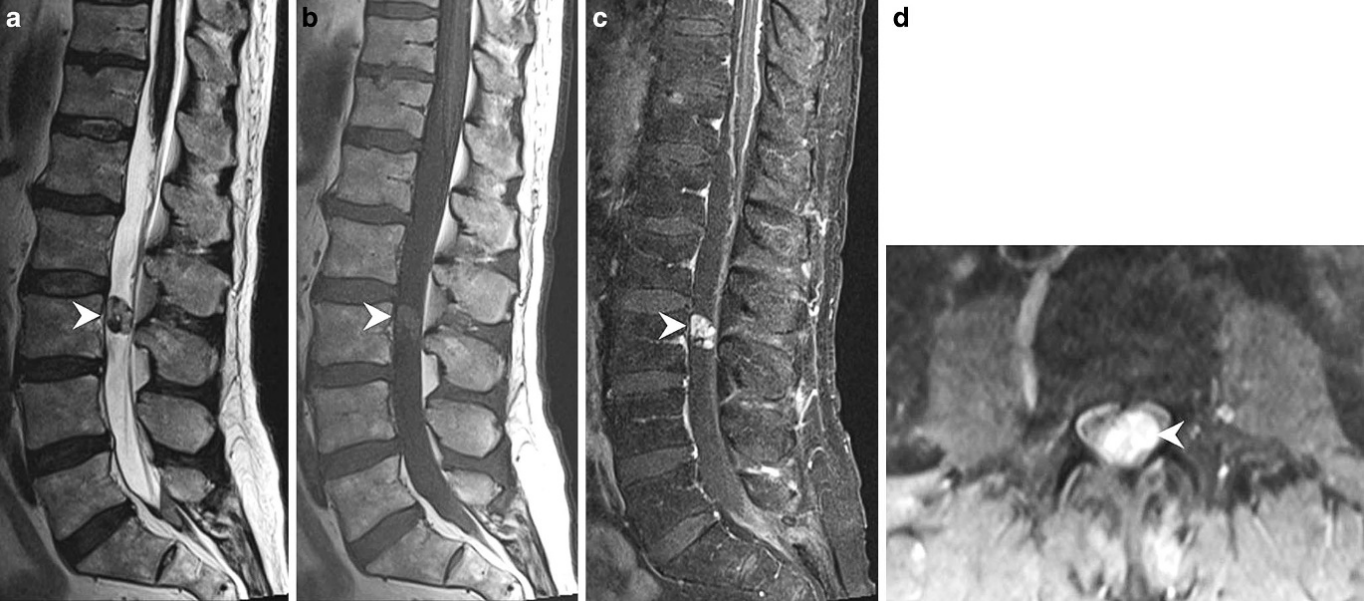
On sagittal T2-weighted images (**a**) a heterogeneous lesion (*arrowhead*) at the L3 level can be appreciated. The lesion shows slightly elevated signal intensities on nonenhanced sagittal T1 weighted images (**b**, *arrowhead*) when compared to cerebrospinal fluid. After administration of gadolinium the lesion shows distinct contrast-enhancement on sagittal (**c**), and axial (**d**) T1-weighted images (*arrowhead*)

Axial diffusion-weighted (b 1000, Fig. 1a) and axial T2-weighted (Fig. 1b) images show a superficial siderosis of the brainstem (Fig. 1a, b, arrowhead) and the cerebellar foliae (Fig. 1a, b, arrow). On sagittal reconstruction of a spinal CT after intrathecal administration of contrast agent (Fig. 2) a dilatation of perimedullary veins (arrow) along the dorsal surface of the myelon through to the conus (arrowhead) is present. Spinal catheter angiography (Fig. 3) injected through the right lumbar artery at the L1 level shows filling of a dilated cauda equina artery (arrow) through a radicular anterior artery as well as a tumor blush (arrowhead) at the L3 level. On sagittal T2-weighted images (Fig. 4a) a heterogeneous lesion (arrowhead) at the L3 level can be appreciated. The lesion shows slightly elevated signal intensities on nonenhanced sagittal T1-weighted images (Fig. 4b, arrowhead) when compared to cerebrospinal fluid. After administration of gadolinium the lesion shows distinct contrast-enhancement on T1-weighted images (Fig. 4c, d, arrowhead).

## Differential Diagnosis

### Myxopapillary Ependymoma

Myxopapillary ependymoma (ME) is the most common primary neoplasm (WHO grade I) of the conus medullaris and cauda equina (83%) [1]. It is considered a subtype of the ordinary ependymoma (13% of all spinal ependymomas) and seems to stem from glial cells of the filum terminale [2]. It peaks between 30–40 years of age. Clinical symptoms can mimic disc herniation and, depending on the size of the tumor, reach a fully developed cauda equina syndrome. Tumor spreading may occur predominantly in the pediatric population [3].

On MRI, the tumor is well delineated with a round or ovoid shape and can be indistinguishable from other tumors. Characteristically MEs occur within a span of 2–4 vertebral segments. They have a lobulated, soft tissue sausage-like appearance that might provoke a bony scalloping or even osseous lytic destruction in the adjacent vertebrae. Due to its rich mucin content it is almost always hyperintense on T2-weighted images and T2-hypointense margins of the tumor can indicate superficial siderosis. Myxopapillary ependymomas have the greatest bleeding tendency of all ependymoma subtypes and may appear with proliferated vessels subsequent to hypervascularity [4]. Usually MEs show avid contrast enhancement. Myxopapillary ependymoma is a valid differential diagnosis in this case.

### Paraganglioma

Paragangliomas are hypervascularized neuroendocrine tumors arising from the neural ectoderm. The spine is a rare site of this slow growing tumor (WHO grade I) with an incidence of 3–4% of all cauda equina tumors [5]. The average age of presentation is 46 years without gender predilection. Clinical symptoms range from back pain to paraparesis/paraplegia [6]. Only 1–3% of paragangliomas have secretory activity with corresponding cardiovascular symptoms, an extremely rare finding in cauda equina paragangliomas [5].

On imaging, the tumor is predominantly encapsulated, heterogeneously hyperintense on T2, sometimes displaying cystic areas indicating previous hemorrhages. Size usually ranges between 10 mm and 50 mm and bony remodeling may be present [7]. Hallmark of the paraganglioma is the hyperperfusion with intense contrast enhancement and flow voids along the surface of the tumor or the spinal cord margins. In addition, superficial siderosis (cap sign) may be present [1]. Repeated subarachnoid hemorrhages have been reported [6].

Paraganglioma of the cauda equina share a great number of imaging features with myxopapillary ependymoma and it can be very difficult to distinguish between the two entities.

### Hemangioblastoma

Spinal hemangioblastomas (HBL) are highly vascularized WHO grade I tumors that can occur sporadically in 20% of spinal hemangioblastomas or present in association with von Hippel-Lindau disease in 80% of cases [8]. Only 8% of all intraspinal HBL have an intradural extramedullary location [9]. Most spinal HBLs arise at the thoracic or cervical level, whereas lumbar or sacral locations are less common. Mean age at presentation is 35 years. Clinical presentation can be unspecific including focal spinal pain or sensory deficits.

The MRI findings can have a wide spectrum of tumor presentations. Small tumors may present with a uniform intense enhancing nodule, predominantly in a subpial or intramedullary location. Larger lesions may show heterogeneous enhancement and tend to have flow voids related to enlarged feeding or draining vessels, indicating hyperperfusion of the lesion. An HBL is associated with syrinx in 40% regardless of the size of the tumor [10]. Spinal cord swelling is another frequent imaging finding. Intradural extramedullar HBLs may resemble meningiomas or schwannomas. The HBLs rarely cause subarachnoid hemorrhages [11]. Presence of multiple spinal HBLs may be indicative von von Hippel-Lindau disease.

In conclusion, the present case has imaging features of intradural extramedullary HBL, but the lack of additional signs and symptoms of a von Hippel-Lindau syndrome makes the diagnosis less likely.

### Metastasis

Intradural extramedullary metastases are very rare and account for 5–10% of all spinal metastases with the lumbosacral region being the most common localization [12]. They most frequently derive from lung and breast cancer, melanoma or lymphoma [13]. In addition, metastases can be associated with primary central nervous system (CNS) neoplasms, such as medulloblastoma, ependymoma or glioblastoma [13]. Clinical symptoms are unspecific typically including back pain. Symptoms are rapid progression and the prognosis is generally poor.

The MRI findings of spinal metastasis vary greatly. A solitary focal round/ovoid mass with strong contrast enhancement is common. Presence of multiple lesions makes the diagnosis more likely. Meningeal spreading, the so-called meningeal carcinomatosis can present with multiple contrast enhancing nodules, a “sugar coating” of the dura/nerve roots or complete filling of the dural sac [13]. Metastases classically are hypervascularized. Metastasis from renal cell cancer, thyroid carcinoma, neuroendocrine carcinoma and melanoma have a higher propensity for spinal hemorrhage.

In patients over 50 years of age, metastases always have to be considered as a potential differential diagnosis. Even though a hypervascularized spinal metastases is a possible cause for subarachnoid blood, a chronic infratentorial siderosis, as in this case, would tend to rule out this diagnosis.

## Histology

### Histology and Immunohistochemistry

**Fig. 5:**
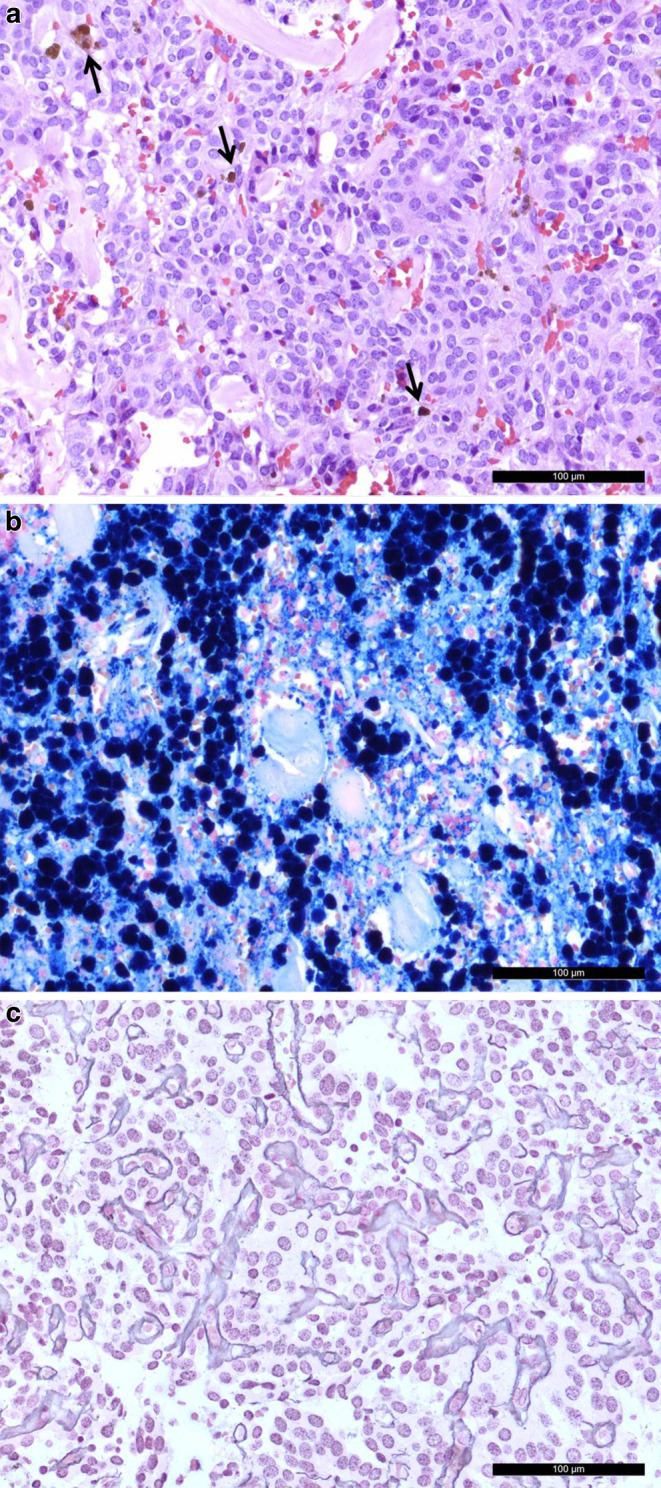
Hematoxylin and eosin (H&E) stain (**a**) shows an isomorphic tumor with a moderate cell density. Fresh bleeding and residuals of older bleeding can be observed (*arrows*). The residuals can also be visualized in a Prussian blue stain (**b**). The tumor cells grow in nest-like structures, so-called lobules or *Zellballen*. Tibor-PAP, a reticulin silver staining (**c**) reveals a delicate reticulin fiber network surrounding these lobules. (Size bars **a–c**: 100 μm)

A specimen of an intradural lesion was obtained for neuropathological analysis. Microscopically, hematoxylin and eosin (H&E) staining revealed a tumor with a moderate density, fresh bleeding and residuals of older bleeding (Fig. 5a, b). The tumor cells grew in nest-like structures, so-called lobules or *Zellballen* (Fig. 5a). These lobules were surrounded by a fine reticulin fiber network (Tibor-PAP stain, Fig. 5c). The cells appeared mostly round and presented with central nuclei with a finely stippled chromatin and a light eosinophilic cytoplasm.

**Fig. 6:**
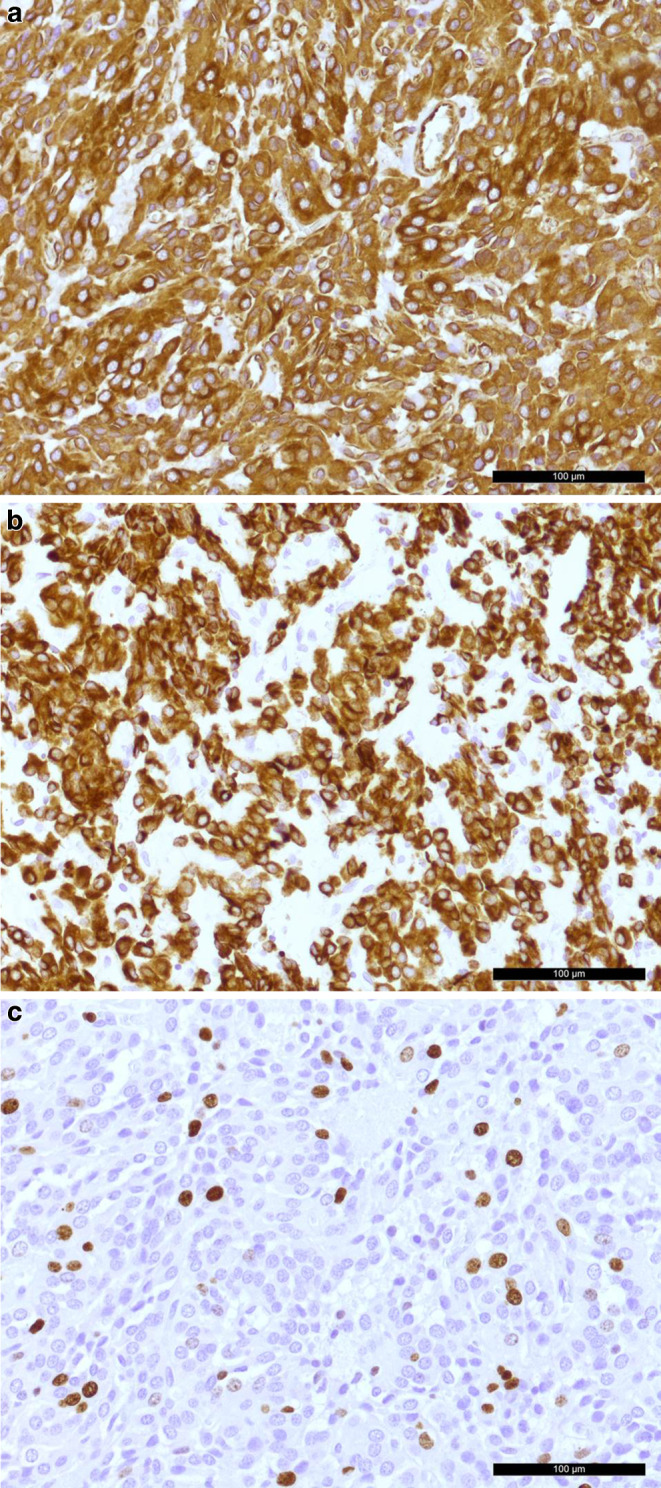
The tumor cells show positivity in the immunohistochemical reaction for vimentin (**a**, *brown*) and the pancytokeratin marker MNF116 (PanCK, **b**, *brown*). The immunohistochemical staining for Ki-67 (**c**) labelled up to 10% of the cells as proliferative (*brown*). All images were taken with a 20× objective. Hematoxylin (*blue*) was used as counterstaining in immunohistochemical reactions. Scale bars represent 100 μm

In immunohistochemical reactions, the cells showed positivity for vimentin (Fig. 6a) and the pancytokeratin marker MNF116 (PanCK, Fig. 6b). Moreover, the cells were partially labelled for CK8/18 (not shown). Positive signal was also observed in the reactions for synaptophysin and partially for neurofilament (both not shown). With up to 10% of the cells labelled by the mitotic marker Ki-67 (Fig. 6c), the proliferation rate was remarkably high. The immunohistochemical reactions for the cytokeratins CK5/6, CK7 and CK20 were negative (not shown). Furthermore, the tumor cells reacted negatively for thyroid transcription factor 1 (TTF1), epithelial membrane antigen (EMA), S100, Olig2, Stat6 and GFAP (not shown).

With PanCK positivity, TTF1 negativity and a neuroendocrine differentiation, a neuroendocrine neoplasm of the digestive system was discussed as a differential diagnosis. We therefore performed additional immunohistochemical stainings for EP4 and CDX2, but only a few tumor cells were labelled by EP4. The reaction for CDX2 remained completely negative. Furthermore, the exclusively intradural tumor manifestation and the characteristics of a paraganglioma (including immunoreactivity for neuroendocrine markers, a lobulated growth pattern and an increased bleeding tendency) speak against the presence of a neuroendocrine tumor. Furthermore, it was shown in recent publications that paragangliomas of the cauda equina are positive for cytokeratin markers [14, 15].

## Diagnosis

### Cauda Equina Paraganglioma (WHO Grade I)

The World Health Organization (WHO) classification of tumors of the CNS defines paraganglioma as a neuroendocrine neoplasm arising in specialized neural crest cells associated with segmental or collateral autonomic ganglia or paraganglia [16]. Paragangliomas typically grow in nest-like structures (*Zellballen*) and show a delicate capillary network. Paraganglioma are graded as WHO grade I. The entity is fairly uncommon in the CNS with the most common CNS localization being the cauda equina/filum terminale. Adults are affected by paragangliomas, with a male predilection. Single paragangliomas are thought to have a benign course. In contrast, multiple paragangliomas or a co-occurrence with other neoplastic processes, e.g. pituitary tumors or renal cancer, imply a genetic predisposition and therefore warrant further investigation [15, 17].
